# Supplementary material for: All-Atomic Molecular Dynamic Studies of Human and Drosophila CDK8: Insights into Their Kinase Domains, the LXXLL Motifs, and Drug Binding Site
Source: Int J Mol Sci. 2020 Oct 12;21(20):7511. doi: 10.3390/ijms21207511 (PMC7590003; doi:10.3390/ijms21207511)

Supplementary Figure 1

a

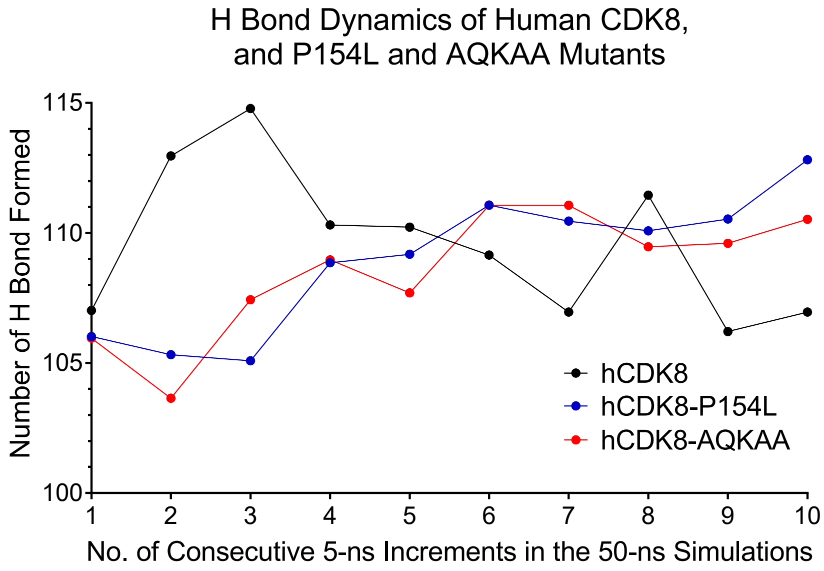

b

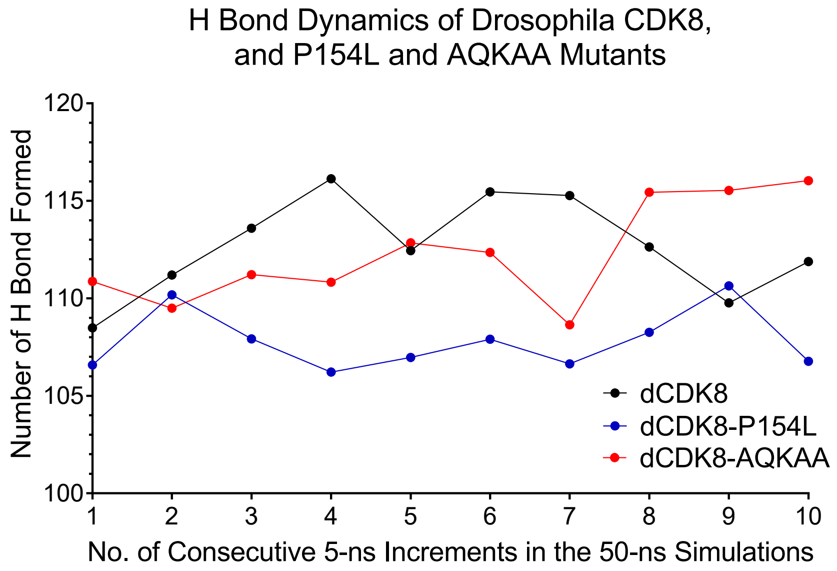

Supplementary Figure 2

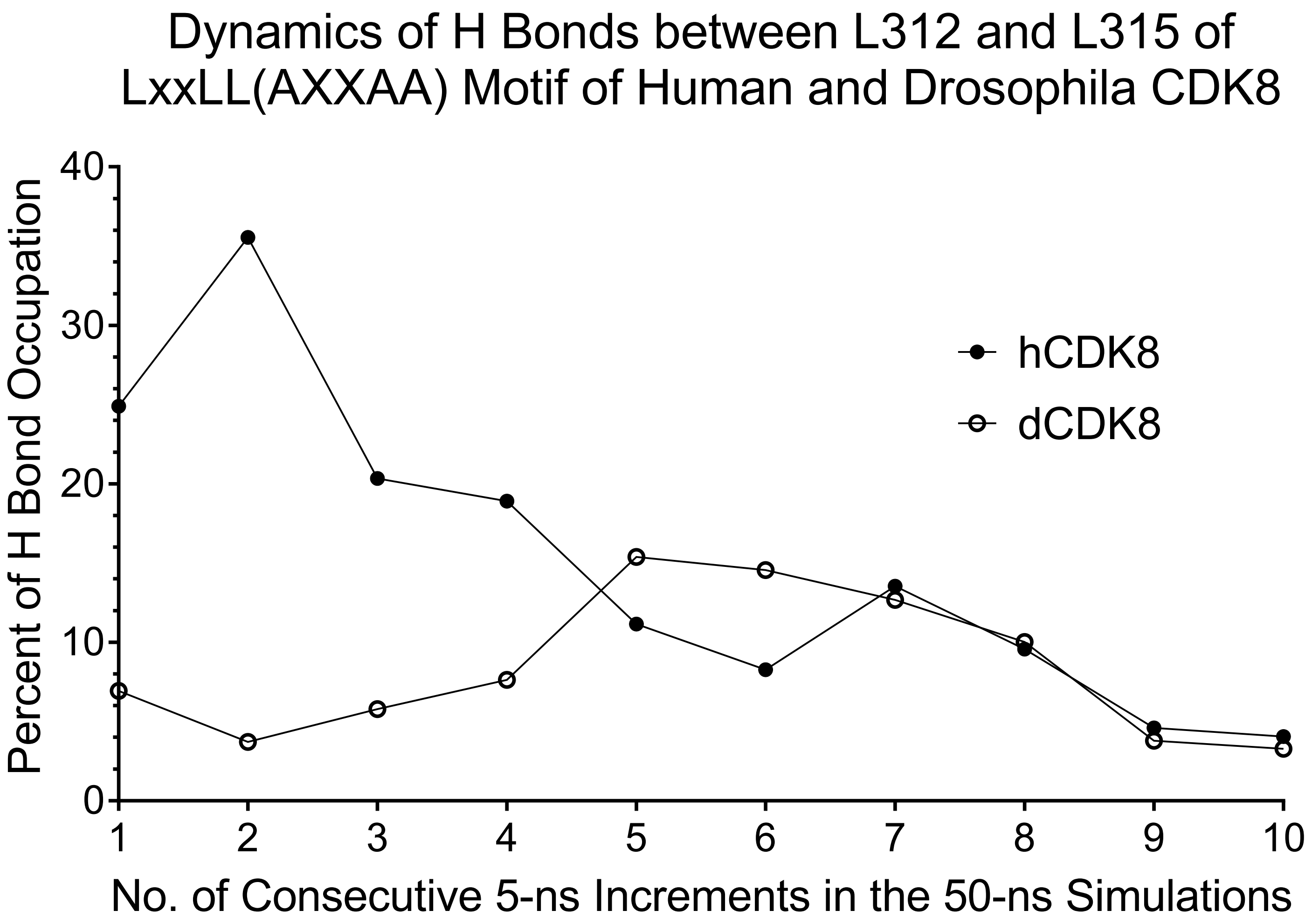

Supplementary Figure 3

a

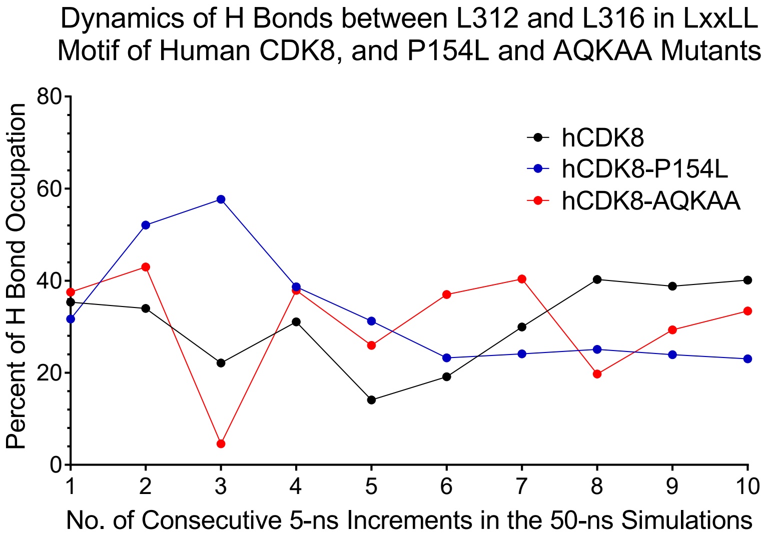

b

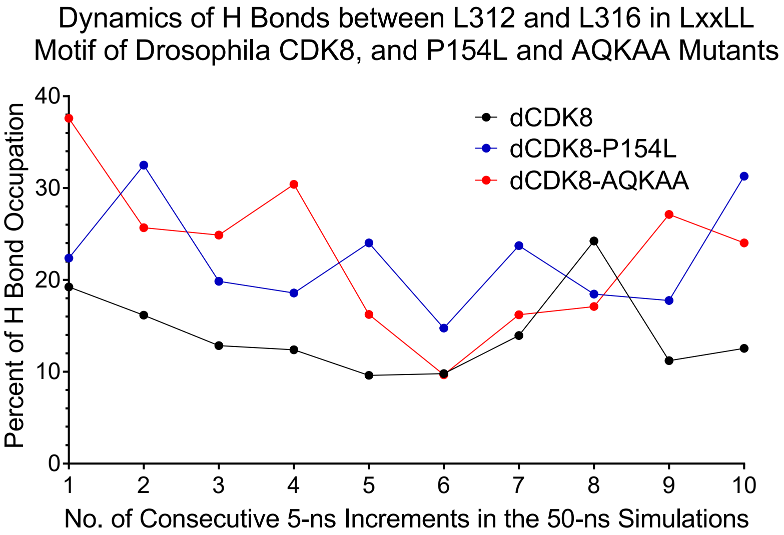

Supplementary Figure 4

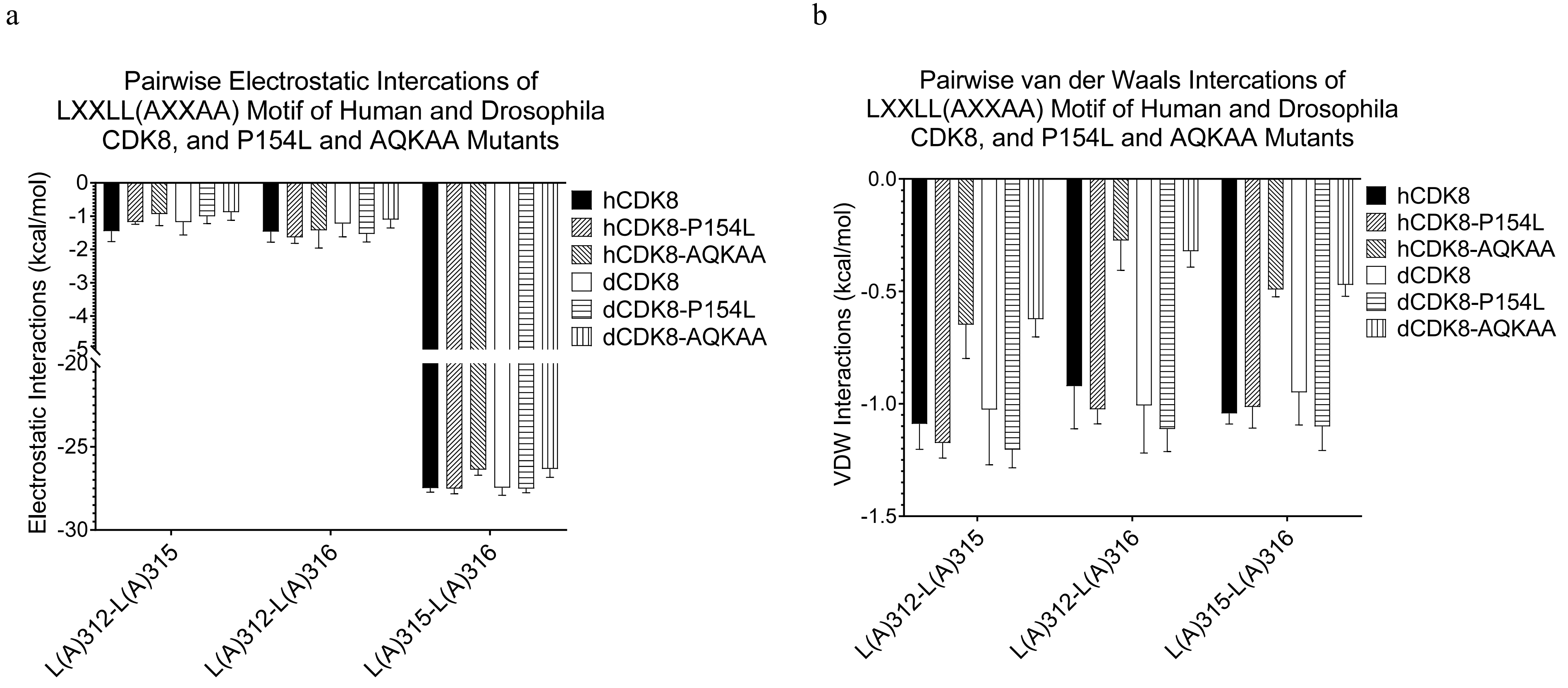

Supplementary Figure 5

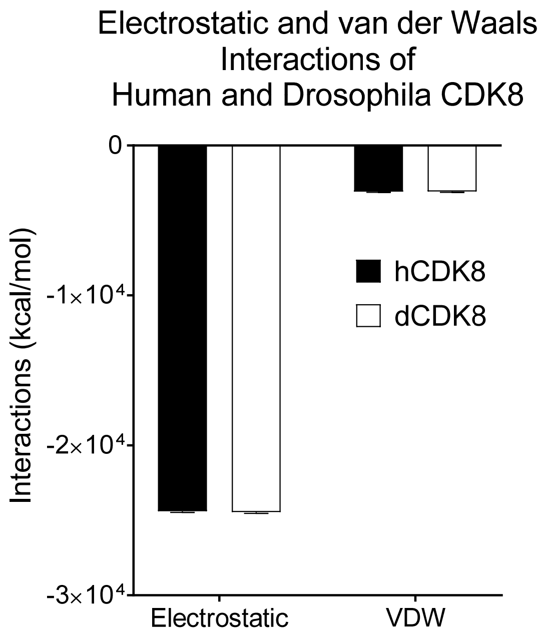

Supplementary Figure 6

a

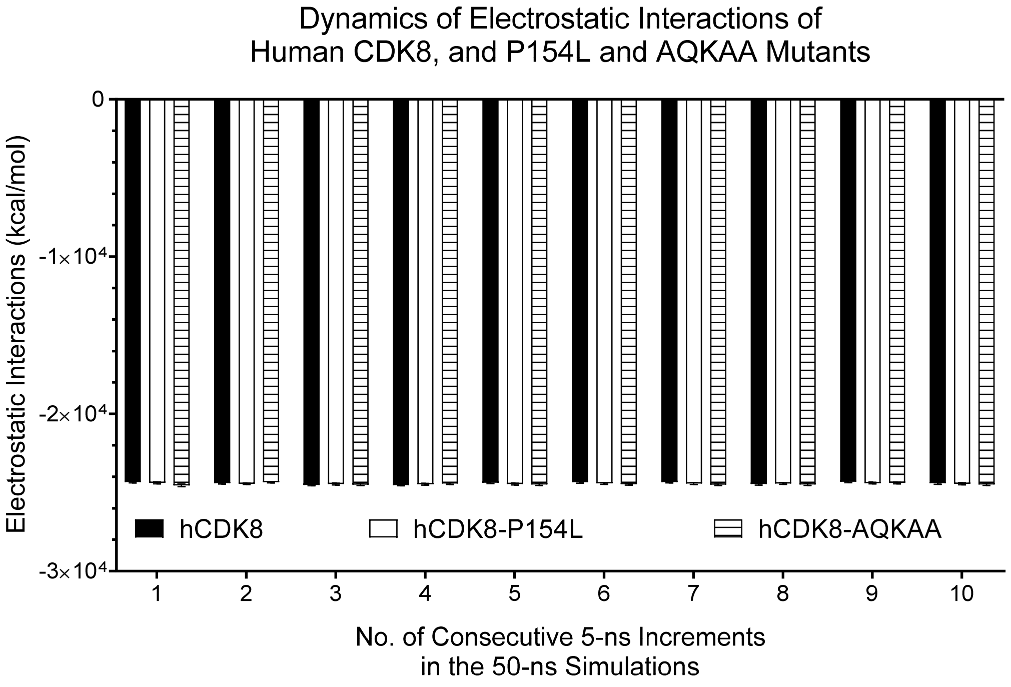

b

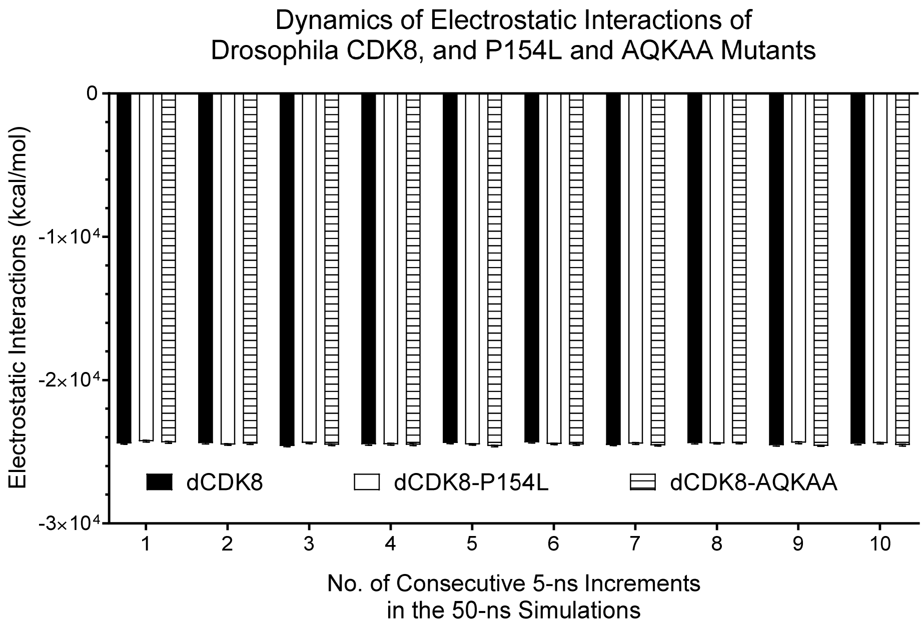

Supplementary Figure 7

a

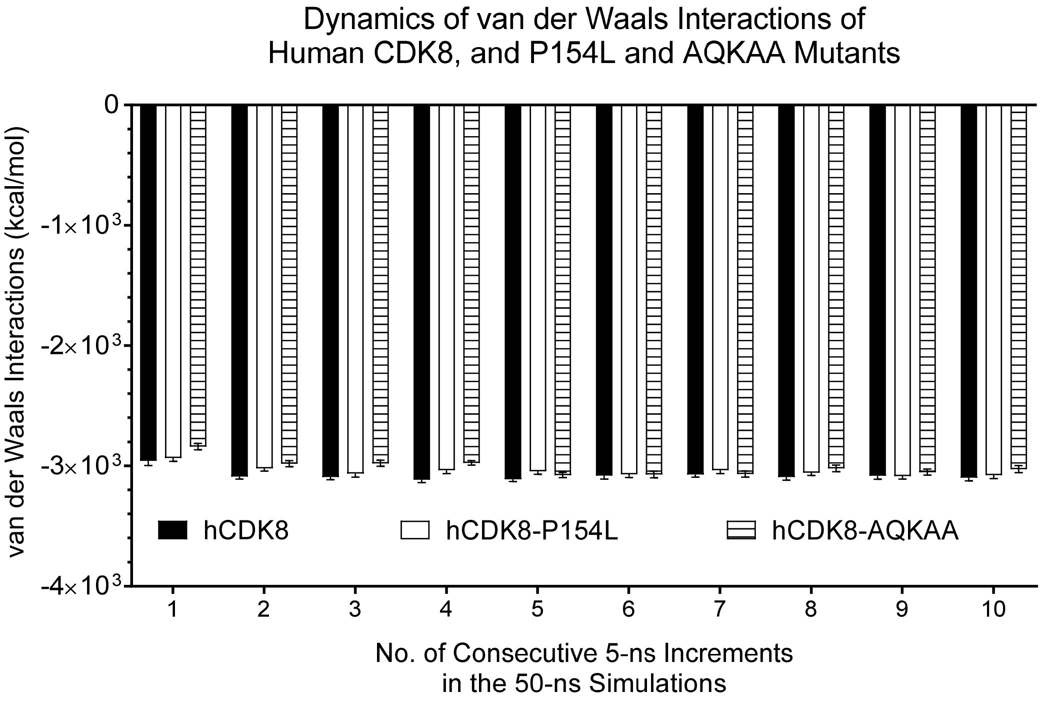

b

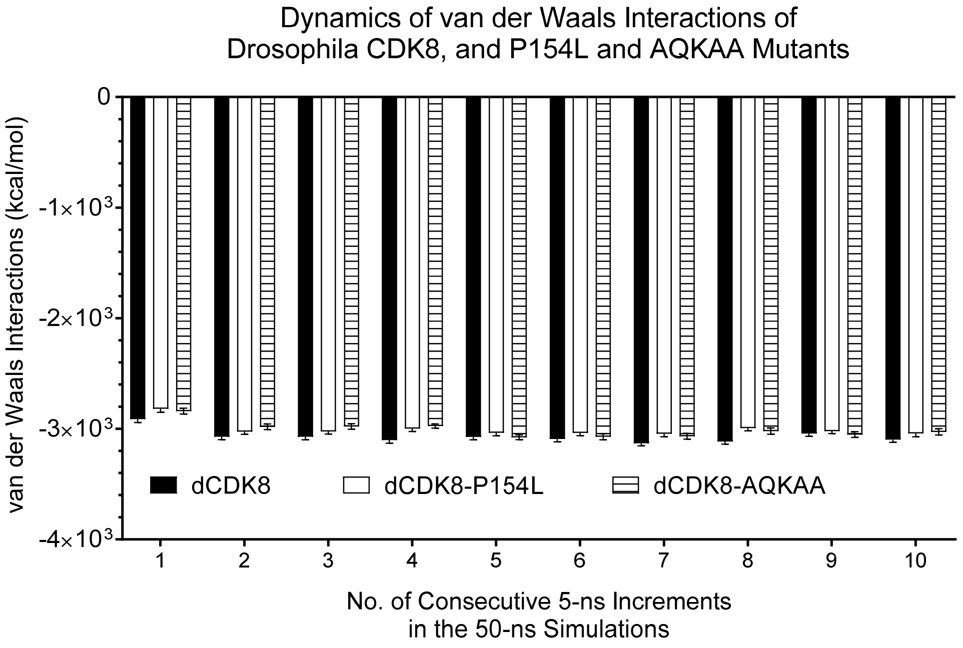

Supplementary Figure 8

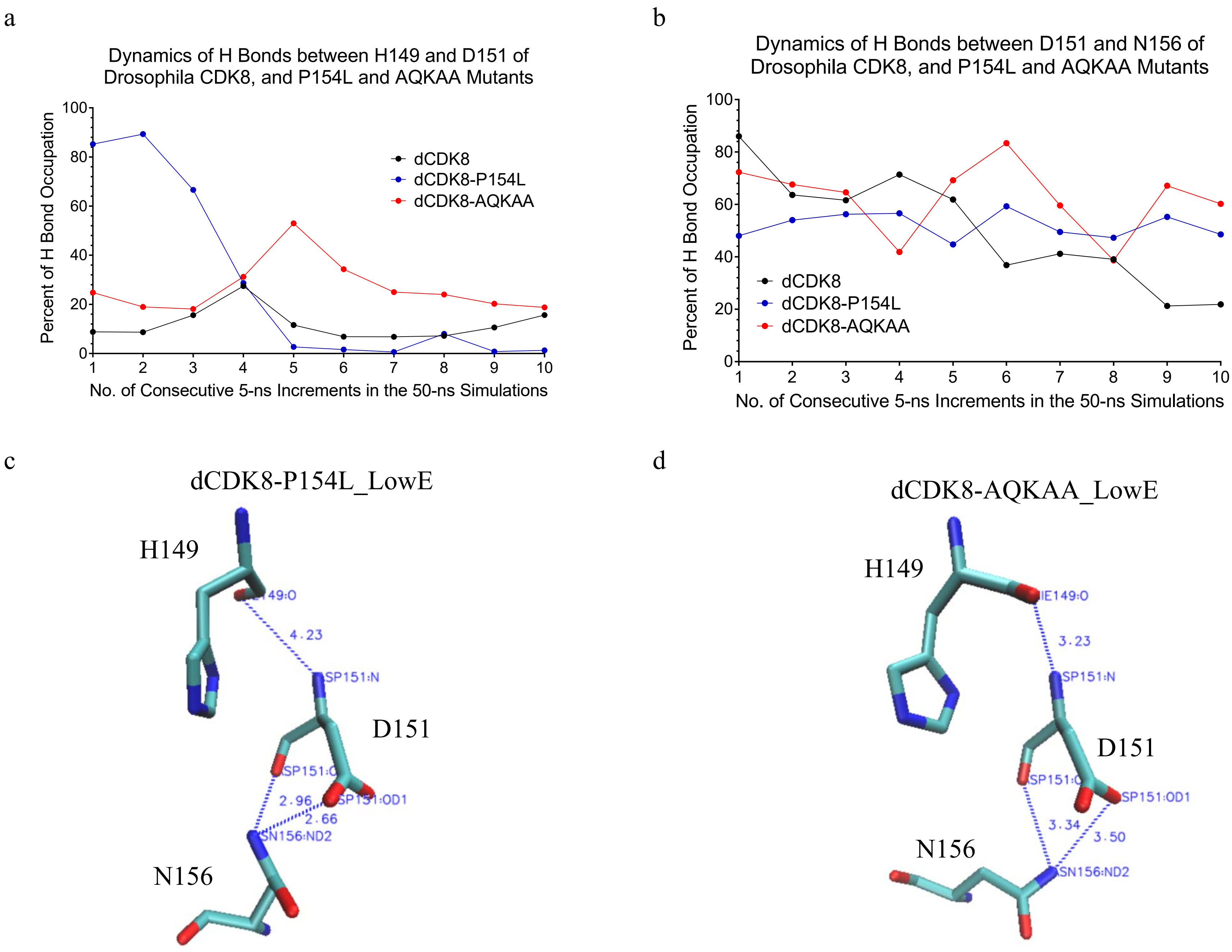

Supplementary Figure 9

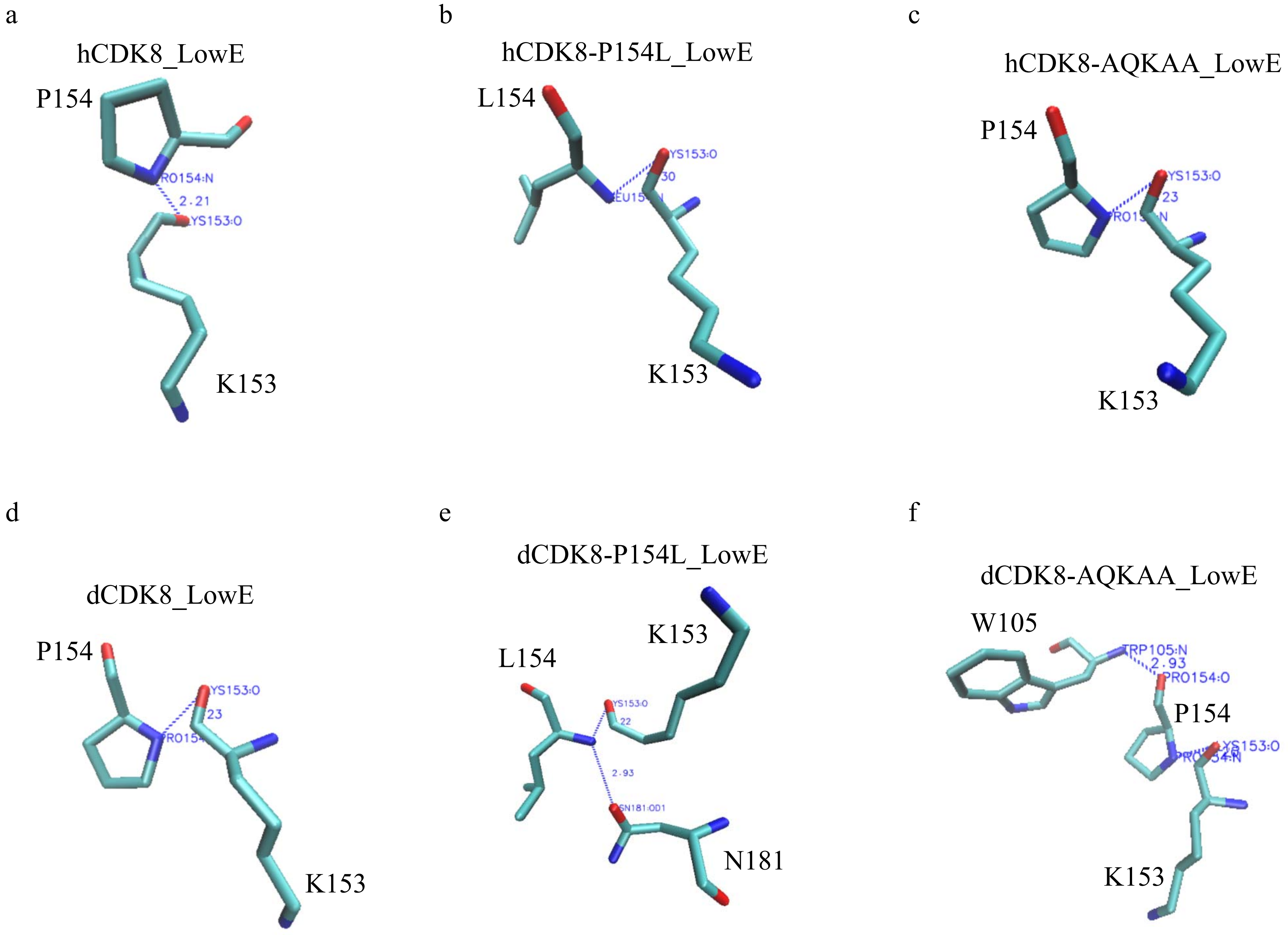

Supplementary Figure 10

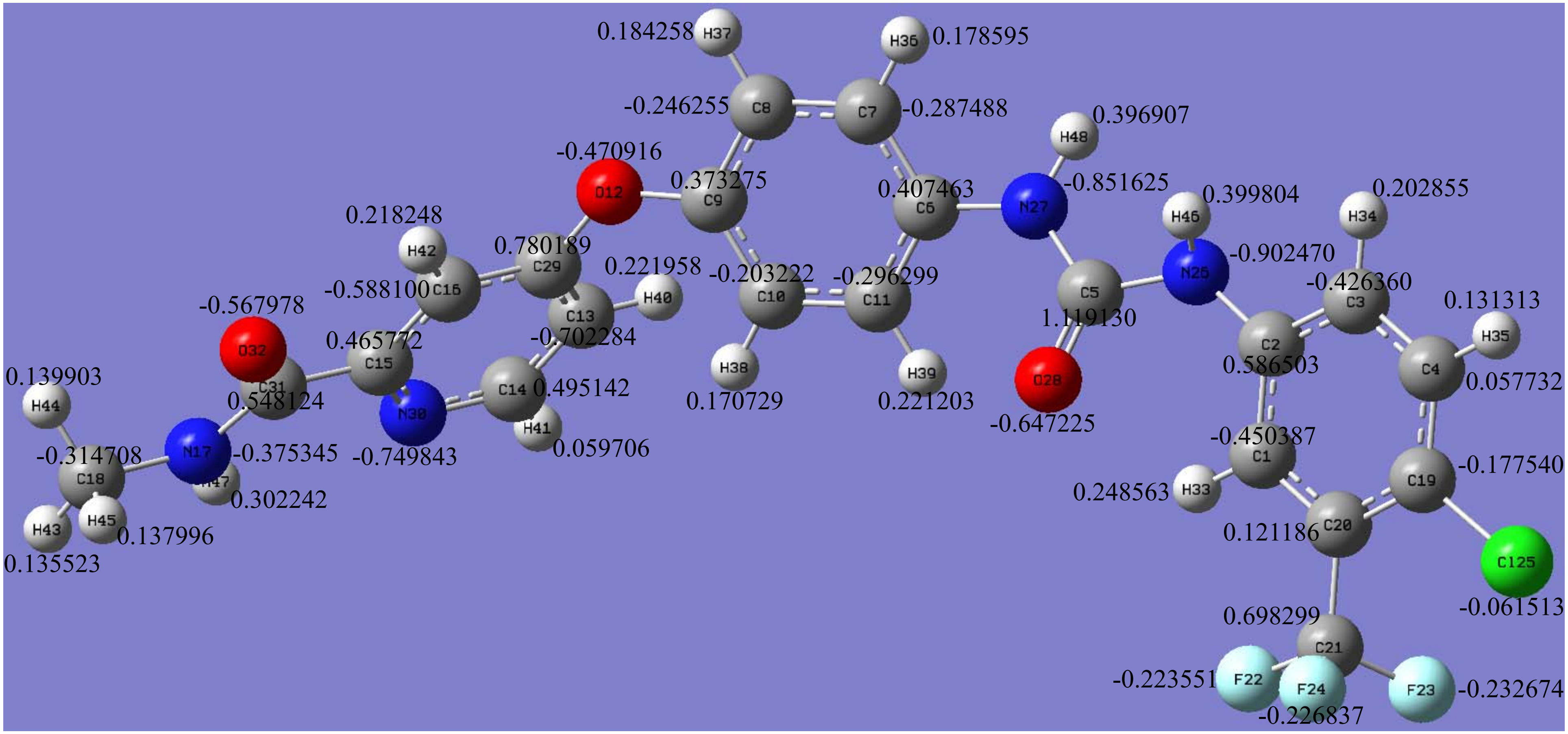

Supplement: Supplementary file 1 [file ijms-21-07511-s001.pdf]
